# Effects of a Video Sequence Based Intervention on Anxiety, Fatigue and Depression in Cancer Patients: Results of a Randomized Controlled Trial

**DOI:** 10.1177/15347354231153172

**Published:** 2023-02-17

**Authors:** Sven Neubert, Sina Schlecht, Karin Meng, Antonia Rabe, Elisabeth Jentschke

**Affiliations:** 1 University of Würzburg, Comprehensive Cancer Center Mainfranken, Würzburg, Germany

**Keywords:** eHealth, psycho-oncology, complementary medicine, psychoeducation, mind-body-intervention, anxiety, depression, fatigue, oncology

## Abstract

**Background:**

Cancer patients often suffer from psychological symptoms and need psychological support. Especially during the COVID-19 pandemic, eHealth interventions might be helpful to overcome the obstacles of the pandemic. This study evaluates the effectiveness of a video sequence-based eHealth intervention on anxiety, fatigue, and depression in cancer patients.

**Methods:**

Patients (N = 157) with different tumor entities were randomly assigned to the video intervention group (IG) and the waiting control group (CG). Patients in the IG received a video intervention comprising 8 video sequences over 4 weeks. The videos included psychoeducation on distress and psychological symptoms, Acceptance and Commitment Therapy elements, and Yoga and Qigong exercises. Patients’ anxiety and fear of progression (primary outcomes) and secondary outcomes were assessed before randomization (T1) and after the end of the intervention for IG or the waiting period for CG (T2) using self-reported questionnaires (GAD-7, PA-F-KF, EORTC QLQ-FA12, PHQ-8).

**Results:**

Patients of the IG showed no significant improvement in anxiety (GAD-7; *P* = .75), fear of progression (FoP-Q-SF; *P* = .29), fatigue (EORTC QLQ-FA12; *P* = .72), and depression (PHQ-8; *P* = .95) compared to patients in the waiting CG. However, symptoms of anxiety, fatigue, and depression decreased in both groups. Exploratory subgroup analysis regarding sex, therapy status, therapy goal, and tumor entity showed no effects. Overall, the intervention had a high level of acceptance.

**Conclusions:**

The video intervention was ineffective in reducing the psychological burden compared to a waiting CG. The findings support prior observations of the value of therapeutic guidance and promoting self-management for improving patients’ psychological burdens. Further studies are required to evaluate the effectiveness of psycho-oncological eHealth delivered through video sequences.

## Introduction

Every year about 500.000 people in Germany develop cancer.^1^ By the end of 2017, there were about 4.65 million people with or after cancer.^2^ Roughly 39% of cancer patients have psychological disorders.^3^ The lifetime prevalence of anxiety disorders for cancer patients was around 25% pre-COVID.^3^ However, the prevalence depended on various influencing factors such as therapy status, sex, age, and tumor entity.^4^ Similar to anxiety, there is a pronounced heterogeneity in the occurrence of fatigue in different patient groups, with an overall average prevalence of about 49% in cancer patients.^5^ Depression also is a common concomitant disease in cancer patients. During the COVID pandemic, depressive symptoms among people with cancer were around 37%.^6^ On the other hand, the prevalence of anxiety^6^ in cancer patients during the pandemic rose to 38% and quality of life deteriorated compared to the average population.^7^ The reported prevalences emphasize the importance of offerings to improve cancer patients’ mental health and well-being.

The COVID-19 pandemic could have worsened these problems,^8^ as it challenges various aspects of life and healthcare, including access to psycho-oncological treatment. Due to the health risks related to direct contact, many patients cannot take advantage of psycho-oncological treatment. However, the demand does not decrease with treatment availability and remains constant during the pandemic. In health psychology, electronic health (eHealth) interventions represent an essential opportunity to actively involve patients in their health care.^9,10^ In 2017 about 69% of cancer survivors searched the internet for information about their disease.^11^ Their high information needs are often unfulfilled due to the high degree of variability in quality, comprehensibility, and accuracy of internet sources.^12,13^ There are promising studies regarding anxiety and depression using eHealth with different psychological interventions.^9,14-17^ However, the results are heterogeneous.^14,15^ Also, studies on fear often only dealt with a few different tumor entities, comprised cancer survivors rather than those in treatment, included relatively few male subjects, and often considered fear of recurrence as the only aspect of anxiety.^9,15-19^ Several meta-analyses have shown promising results from eHealth interventions on fatigue,^9,20^ but further randomized controlled trials (RCTs) are required to confirm these results.^20^ Due to the lack of eHealth studies for a heterogeneous sample of cancer patients and survivors, we aimed to create an eHealth intervention for a broad range of cancer patients to benefit from a remotely-accessible and widely applicable psycho-oncological offering during the COVID19-pandemic. To this end, we employed various psychological methods, including psychoeducation and Acceptance and Commitment Therapy elements. These methods have been shown to reduce anxiety,^21-26^ depression,^21,22,25-29^ and fatigue.^30-33^ Additionally, we included complementary and alternative medicine elements that affected the target variables, such as Yoga^34-37^ and Qigong.^38,39^ Furthermore, we selected the techniques we already used in psycho-oncological routine care.

We hypothesized:

(1) The participants in the video sequence-based IG will have significantly more improved anxiety levels and fear of progression than the participants in the waiting CG after the end of therapy (primary outcome).
(2a) The participants in the video sequence-based IG will have significantly more improved values for fatigue after the end of therapy than the participants in the waiting CG.
(2b) The participants in the video sequence-based IG will have significantly more improved values for depression after the end of therapy than the participants in the waiting CG.

Moreover, we investigated the following exploratory questions regarding the further implementation of digital psycho-oncological offers for cancer patients within the acute care setting.

Are there certain patient groups who benefit more from the intervention than others, regarding sex, therapy status, therapy goal and tumor entity?

Does therapy adherence have an effect on the outcomes within the IG?

## Methods

### Trial Design

The study was a single-center, prospective, randomized, controlled intervention study with a waiting CG performed at the University Hospital of Wuerzburg, Comprehensive Cancer Center Mainfranken (CCCMF). The Ethics Committee of the University of Würzburg approved the study on 23.04.2021 (Nr. 123/20-me).

Cancer patients with diverse tumor entities and acute appointments in institutions of the CCCMF (interdisciplinary oncological therapy outpatient clinic (IOT), various oncological stations of the University Hospital Wuerzburg, and ambulatory psycho-oncology patients) were evaluated by medical records. Eligible patients were contacted on site or by telephone and asked to participate in the study. Afterward, we sent a written patient information sheet and consent form to interested patients contacted by telephone. After signing the consent form and completing the first questionnaire to collect the baseline values (T1), participants were randomly assigned to the IG or CG using a computer-generated list of random numbers. We used a randomization procedure with an allocation ratio of 1:1. A list of participants’ anonymous study numbers was used for external randomization. A scientific member of the Institute of Clinical Epidemiology, University of Wuerzburg, performed randomization (central randomization per envelope), guaranteeing allocation concealment.

Then the participants of the IG received the video intervention for 4 weeks, while the participants of the CG had a 4-week waiting period and did not receive any comparable therapy. Both groups completed another questionnaire at the end of this period (T2). After the post-intervention survey, the CG also received a 4-week intervention.

Sample size was powered to detect a medium-sized between-group effect (*d* = 0.5, 2-sided, α = .05, 1-β = .8) in the primary outcome. Therefore, 128 persons are required. Concerning a possible drop-out, we recruited a sample of N = 172 patients.

### Participants

**Figure 1.**
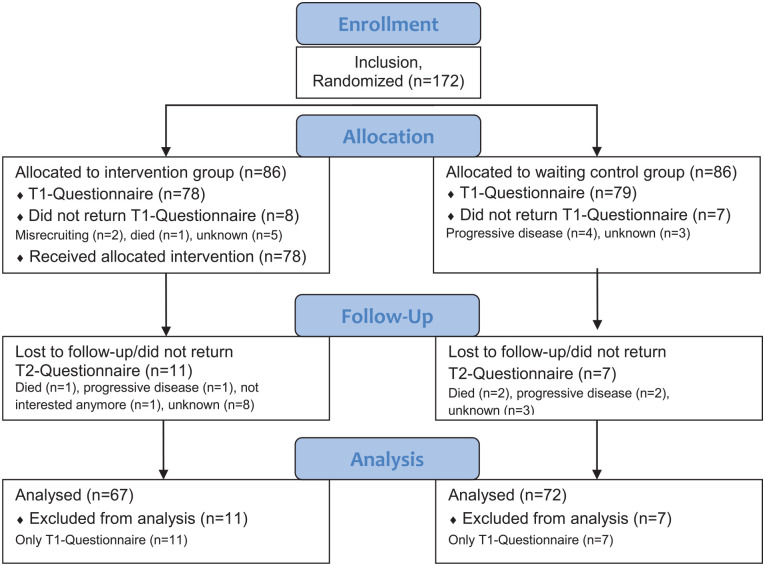
Patient flow

The inclusion criteria were a malignant tumor disease in the history of the patient, a minimum age of 18 years, and informed consent to participate in the study. There was no preselection regarding the current stress level. Exclusion criteria were insufficient German language ability and severe physical or mental impairments. The patients were recruited from June to September 2020 within the CCCMF facilities. Figure 1 shows the participant flow.

### Intervention

Overall, the intervention comprised 8 videos, each about 10 to a maximum of 30 minutes in length. The structure of all units was similar. Each sequence started with imparting knowledge on the respective topic. There was both an explanation of the backgrounds and meanings of the respective symptoms and assistance using elements of different psycho-oncological tools in order to be able to manage symptoms. The psychoeducation was supported in each case by text slides and illustrations. Yoga, relaxation, or Qigong exercises were implemented at the end of each video. Two ACT core processes (ie, contact with the present moment and defusion) are addressed through parts of the education and exercises in specific video sequences. An experienced psycho-oncologists/psychotherapist who is also a certified yoga teacher performed the video intervention (7 out of 8 sequences). The Qigong sequence was administered by an experienced psycho-oncologist/psychotherapist who is also a qualified Qigong instructor. The yoga exercises were already evaluated in previous trials.^35,40^ Detailed descriptions and illustrations of the exercises from the videos can be found in “Heilkraft Yoga: 100 Übungen für Ihre Gesundheit” by Sigmund Feuerabendt.^41^ However, for details on the individual sequences, see Table 1.

**Table 1.** Video Sequences.

Sequence (duration)	Content
1: Fear (15 min)	Psychoeducation: Fear as a natural protective mechanism, fear of recurrence and progression, concern in the family system, hidden burdens, common patient fears, psychoneuroimmunology, fear-stress reaction system, importance of breathing in fear. Yoga exercise (“breathing circle”)
2: Mind control (12 min)	Psychoeducation: Introduction on psychological approaches (Cognitive-behavioral therapie, Acceptance and Commitment Therapy), relationship between thoughts and emotions, questioning negative thoughts, cognitive restructuring from behavior therapy, mind control and cognitive defusion task, reflection of thoughts. Yoga exercise (“the warrior”)
3: Fatigue 1 (18 min)	Psychoeducation: Tumor-associated fatigue, dimensions of fatigue; physical fatigue, emotional fatigue, cognitive fatigue, causes of fatigue, possible interventions. Yoga exercise (“standing with the scales”)
4: Fatigue 2 (18 min)	Psychoeducation: Energy management, incorporating rewards into everyday life, positive influence on the body, slow increase in energy consumption. Yoga exercise (“the venous pump”)
5: Resilience (20 min)	Psychoeducation: Multifactorial development of cancer, exhausting and stressful thoughts, ABC model by Albert Ellis, resilience research, critical questioning of thoughts, pillars of resilience, assessment of situations. Yoga exercise (“the tree”)
6: Healthy sleep (17 min)	Psychoeducation: Theory of sleep, sleep-promoting factors, sleep diary, thought circles, dysfunctional thoughts, mind control, dealing with stressful thoughts Relaxation exercises: breathing exercise, shoulder stretching
7: Qigong (12 min)	Psychoeducation: Introduction to Qigong, integration into everyday life, origin, goals of Qigong, elements of Qigong exercise (“five elements”)
8: Relaxation (33 min)	Psychoeducation: Temple of health (Essen model), pillars of health, interaction of the individual pillars, importance of relaxation, visualized images, summary of the interventions, possibilities of help through therapy. Relaxation exercise (“body scan,” “calming place”)

The participants were given access to a website, on which 2 video sequences were provided weekly for 4 weeks. The patients could watch them via desktop/laptop or tablet as often as they wanted and save the videos and a summary of the exercises. Participants were notified by email each time new videos were available. Technical problems could be announced to an assistant, but problems were rare.

### Measures

#### Outcomes were assessed using self-report validated questionnaires

The Generalized Anxiety Disorder-7 questionnaire (GAD-7) was used to record *anxiety symptoms.*^42^ The questionnaire was also validated for cancer patients.^43^ Items on anxiety symptoms within the last 2 weeks are answered on a 4-point Likert scale (0 = not at all; 1 = on individual days; 2 = more than half the days; 3 = nearly every day). Items are summed-up to a general anxiety score (range: 0-21), with higher scores representing more anxiety.

Furthermore, to assess *fear of progression*, the short form of the Fear of Progression Questionnaire (FoP-Q-SF^44,45^) was administered.^46^ Patients rate 12 items on a 5-point Likert scale (1 = never to 5 = very often). Items are summarized to a sum score (range: 0-60), with higher scores indicating higher fear of progression.

We used the European Organization of Research and Treatment of Cancer Quality of Life Questionnaire-Fatigue 12 questionnaire (EORTC QLQ-FA12) to determine *cancer-related fatigue.*^47^ Twelve items comprising fatigue’s physical, cognitive, and emotional aspects are rated on a 4-point Likert scale. The total score is transformed to a scale ranging from 0 to 100, with higher scores representing more severe fatigue symptoms.

Finally, the Patient Health Questionnaire-8 (PHQ-8) was used to measure *depressive symptoms.*^48^ Patients rate 8 items that refer to symptoms of a depressive episode during the last 2 weeks on a 4-point Likert scale (0 = not at all to 3 = nearly every day). Items are summed up to a sum score ranging from 0 to 24, with higher values indicating more severe depressive symptoms. The PHQ-8 was selected instead of the Patient Health Questionnaire-9, as this has proven to be superior for e-mail, internet, or telephone contact.^49^

*Participants’ satisfaction* with the video intervention and adherence to therapy was assessed using a self-created questionnaire based on further studies.^35^ Patients were asked to judge the content of the video sequences, their usefulness, and the selection of exercises on a 6-point scale (1 = very good, 6 = insufficient). In addition, 3 items inquired about the general satisfaction with the program. Furthermore, patients were asked about video use and training using several categorical items.

### Statistical Analysis

For the analysis, the differences in the outcomes of T1 and T2 were calculated to retain the target variable change. These changes were compared between IG and CG. Questionnaires with missing values were removed from the evaluation. Thus, outcomes were analyzed by pair-wise deletion. Shapiro-Wilk and Levene tests were used to test for normality and homogeneity. Wilcoxon Rank Sum Tests for independent samples were used where the assumptions were not met. Furthermore, we explored intervention effects within specific subgroups, that is, gender (female, male), therapy status (currently in therapy, currently not in therapy), therapy goal (curative, palliative), and the 2 most common tumor entities (hemato-oncological malignancies (hem), breast cancer (bc)). Additionally, the influence of therapy adherence on the outcome was explored within the IG with unpaired 2-sample Wilcoxon Rank Sum Tests comparing the outcomes of participants who watched all the video sequences and participants who watched only part of the videos. All statistical analysis was performed using IBM SPSS Statistics (International Business Machines Corporation Statistical Package for Social Sciences), Version 26, R (Software Version 4.1.1), and RStudio (Version 1.4.1717).

## Results

### Sample Description

Table 2 shows the demographic and clinical sample characteristics of the participants by study group. 69% of the participants were female, and the mean age was 56 years (SD = 12.4). The most common tumor entities among the patients were hem (32%) and breast cancer (30%). 59% of the patients received neoadjuvant or adjuvant therapy during the study period. About 20% had a palliative treatment goal.

**Table 2.** Demographic and Clinical Characteristics of the Sample by Group.

Characteristics	All (n = 157)	IG (n = 78)	CG (n = 79)
Age in years, mean (SD); range	55.5 (12.4); 20-82	55.2 (12.2); 23-77	55.8 (12.7); 20-82
Sex, n (%)			
Female	108 (68.8)	56 (71.8)	52 (65.8)
Male	49 (31.2)	22 (28.2)	27 (34.2)
Tumor entity, n (%)			
Hemato-oncological malignancies	50 (31.8)	19 (24.4)	31 (39.2)
Breast cancer (bc)	47 (29.9)	25 (32.1)	22 (27.8)
Gynecological cancer other than bc	13 (8.3)	6 (7.7)	7 (8.9)
Gastrointestinal cancer	20 (12.6)	9 (11.6)	11 (13.9)
Skin cancer	6 (3.8)	6 (7.7)	0 (0.0)
Malignant head and neck tumors	4 (2.5)	3 (3.8)	1 (1.3)
Pancreatic cancer	4 (2.5)	2 (2.6)	2 (2.5)
Lung cancer	3 (1.9)	1 (1.3)	2 (2.5)
CNS malignancies	3 (1.9)	2 (2.6)	1 (1.3)
Gallbladder cancer	2 (1.3)	2 (2.6)	0 (0.0)
Other	5 (3.2)	3 (3.8)	2 (2.5)
Therapy during study, n (%)^a^			
Any therapy	93 (59.2)	46 (59.0)	47 (59.5)
Chemotherapy	54 (34.4)	21 (27.0)	33 (41.8)
Radiation therapy	11 (7.0)	9 (11.5)	2 (2.5)
Antibody therapy	33 (21.0)	14 (17.9)	19 (24.1)
Hormone therapy	14 (8.9)	10 (12.8)	4 (5.1)
Unknown	2 (1.3)	1 (1.3)	1 (1.3)
Treatment intention, n (%)			
Curative	106 (67.5)	53 (67.9)	53 (67.1)
Palliative	32 (20.4)	15 (19.2)	17 (21.5)
Unknown	19 (12.1)	10 (12.8)	9 (11.4)

Abbreviations: n, number of patients; IG, intervention group, CG; control group; SD, standard deviation.

a Multiple therapies possible.

### Intervention Adherence and Evaluation

The intervention had a high level of acceptance as 93.9% stated that their initial expectations regarding the intervention were at least more likely to be fulfilled and 88.2% stated they would be at least likely to participate in such an intervention again, and 98.5% would recommend it to other patients. Mean ratings of content, selection of exercises, and the usefulness of the sequences were good (Table 3). 83% of the IG participants stated that they had watched all video sequences at least once during the intervention. There was no significant impact on the outcome whether participants watched all video sequences or not (data not shown).

**Table 3.** Evaluation and Use of the Video Sequences.

Video sequences	n	Mean (SD)
Content of sequence		
1: Fear	66	1.41 (0.76)
2: Mind control	66	1.50 (0.77)
3: Fatigue 1	61	1.54 (0.83)
4: Fatigue 2	61	1.60 (0.82)
5: Resilience	62	1.55 (0.80)
6: Healthy sleep	63	1.56 (0.80)
7: Qigong	63	2.0 (1.05)
8: Relaxation	63	1.54 (0.93)
Selection and combination of exercises	52	1.62 (0.89)
Usefulness for coping with the disease	65	1.89 (0.95)
Use and adherence	n (%)	
Watched all video sequences	55 (83)	
Watched video sequences multiple times		
≤2 times0	34 (51)	
3-5 times	10 (15)	
>5 times	1 (2)	
Exercised during the intervention		
No	5 (8)	
Partly	28 (42)	
1-2 times a week	19 (28)	
3-5 times a week	13 (19)	
Daily	2 (3)	
Planned to continue exercising after the end of the intervention		
No	4 (6)	
Partly	33 (49)	
Yes	30 (45)	

Abbreviations: n, number of patients; SD, standard deviation.

### Intervention Effects on Primary and Secondary Outcomes

Table 4 presents results on primary and secondary outcomes, and Supplemental Table 1 shows the exploratory subgroup analysis results.

**Table 4.** Effects on Primary and Secondary Outcomes.

	n	T1 IG mean (SD)	T2 IG mean (SD)	Change IG mean (SD)	n	T1 CG mean (SD)	T2 CG mean (SD)	Change CG mean (SD)	W	*P*-value
GAD-7	67	6.61 (4.58)	5.72 (4.26)	0.90 (3.14)	68	7.00 (5.12)	6.00 (4.50)	1.00 (3.71)	2350	.751
FoP-Q-SF	56	32.43 (9.08)	31.91 (9.99)	0.52 (6.38)	60	33.63 (10.30)	31.97 (9.68)	1.67 (5.16)	1487	.286
EORTC QLQ-FA12	62	36.78 (21.73)	34.50 (24.65)	2.28 (19.66)	70	40.60 (23.83)	37.06 (21.43)	3.53 (17.70)	2089.5	.715
PHQ-8	60	6.97 (4.62)	5.95 (4.85)	1.02 (3.60)	65	7.69 (5.15)	6.78 (4.60)	0.91 (3.56)	1938	.954

Abbreviations: n, number of patients; T1, baseline; T2, after the intervention or waiting period; IG, intervention group, CG; control group; SD, standard deviation.

*Primary outcome*: There were no significant between-group differences in the mean change in anxiety (GAD-7) and fear of progression values (FoP-Q-SF) from T1 to T2 between IG and CG. In both IG and CG, there was a reduction in anxiety and fear of progression from T1 to T2.

*Secondary outcomes*: IG and CG showed no significant differences in the mean change in fatigue (EORTC QLQ-FA12) or depression (PHQ8), respectively. Both groups showed a reduction in the outcomes between baseline values and after the video sequence period.

Exploratory subgroup analysis concerning sex, therapy goal, therapy status, and tumor entity showed no significant between-group differences in the primary and secondary outcomes.

## Discussion

This study showed no improvement in anxiety, fatigue, and depression after a 4-week eHealth intervention in video sequences compared to a waiting CG. A follow-up study 3 months after the end of the intervention will examine the possible long-term changes. Though the results of IG and CG did not significantly differ, there was a high recommendation rate of the intervention among the participants of the IG. Unlike most other studies in this field, this study included a very heterogeneous population, which corresponds to the need for studies on a broader range of cancer patients.^50^ Participants were not pre-selected regarding their initial symptom burden compared to another study that used similar yoga exercises.^40^ Another difference from previous studies is the digital setting. Patients cannot benefit from interaction with a group leader or a possible group effect as they could in the previous studies using similar exercises.^35,40^ Compared to other, more effective interventions, it is noticeable that professional support is often associated with better outcomes.^15,51^ Results fit with studies that found no effect with an eHealth intervention without professional support.^9,18^ Since this study aimed to reach as many patients as possible without the risk of infection and as the participants should incorporate the intervention individually into their daily routine, video sequences were the medium of choice. This study supports the importance of the interpersonal aspect in psycho-oncology. Given the decay in eHealth use over time,^52^ we designed the study with a brief duration. Longer-duration interventions seem to be more effective.^53^ Overall, the literature on eHealth interventions is still very heterogeneous,^14^ and there is a great need for research in this area. Future studies should include innovative adaptive designs to create personalized psychosocial eHealth interventions.^19,54^

The study has several limitations that need to be considered. First, the trial had a waiting-only CG that received no treatment. Participants were not blind to the allocated intervention. Second, there was no possibility of controlling intervention adherence as the participants watched the videos at home. Third, the results of this trial are not generalizable to all tumor entities. However, we included patients with subjective needs for psycho-oncological support. Fourth, we conducted no screening concerning psychological burden. More significant changes might have been achieved in cancer patients with higher symptom severity. Finally, we used different therapeutic tools to create the intervention. Therefore, it is impossible to determine which of the methods used have the highest potential to reduce the symptom burden for each objective in each patient.

## Conclusions

The intervention could not improve the anxiety, fear of progression, fatigue, or depression compared to the waiting CG. However, both groups showed decreased symptoms during the intervention period. In addition, there was high satisfaction and adherence with the intervention among the participants of the IG. Hence, our findings support the observation that more interactive therapeutic guidance and self-management tools might be necessary to improve the impact on patients’ mental health. Thus, further studies are required to evaluate the effectiveness of eHealth delivered through video sequences. Those studies might focus on only 1 or 2 of the therapeutic elements used in this trial to increase the results’ generalizability and informative value. This trial can serve as a further step toward the development of a digital model for the delivery of psycho-oncologic content that is highly scalable, widely disseminable at low cost, and works regardless of the pandemic situation while giving a direction for future targeted eHealth interventions for the management of the examined target variables.

## Supplemental Material

sj-docx-1-ict-10.1177_15347354231153172 – Supplemental material for Effects of a Video Sequence Based Intervention on Anxiety, Fatigue and Depression in Cancer Patients: Results of a Randomized Controlled Trial Click here for additional data file. Supplemental material, sj-docx-1-ict-10.1177_15347354231153172 for Effects of a Video Sequence Based Intervention on Anxiety, Fatigue and Depression in Cancer Patients: Results of a Randomized Controlled Trial by Sven Neubert, Sina Schlecht, Karin Meng, Antonia Rabe and Elisabeth Jentschke in Integrative Cancer Therapies

## References

[1] Kowollik R Buttmann-Schweiger N. Krebs in Deutschland für 2015/2016. 12. Ausgabe. Robert-Koch-Institut; 2019.

[2] Arndt V Dahm S Kraywinkel K. Cancer prevalence in Germany in 2017 Number of cancer survivors based on data from population-based cancer registries. Onkologe. 2021;27:717-723.

[3] Kuhnt S Brähler E Faller H , et al. Twelve-month and lifetime prevalence of mental disorders in cancer patients. Psychother Psychosom. 2016;85:289-296. 27508418 10.1159/000446991

[4] Sigorski D Sobczuk P Osmola M , et al. Impact of COVID-19 on anxiety levels among patients with cancer actively treated with systemic therapy. ESMO Open. 2020;5:e000970. 10.1136/esmoopen-2020-000970 PMC7590347 33097653

[5] Al Maqbali M Al Sinani M Al Naamani Z Al Badi K Tanash MI . Prevalence of fatigue in patients with cancer: a systematic review and meta-analysis. J Pain Symptom Manag. 2021;61:167.e14-189.e14. 10.1016/j.jpainsymman.2020.07.037 32768552

[6] Ayubi E Bashirian S Khazaei S. Depression and anxiety among patients with cancer during COVID-19 pandemic: a systematic review and meta-analysis. J Gastrointest Cancer. 2021;52:499-507. 33950368 10.1007/s12029-021-00643-9 PMC8096890

[7] Ciążyńska M Pabianek M Szczepaniak K , et al. Quality of life of cancer patients during coronavirus disease (COVID-19) pandemic. Psychooncology. 2020;29:1377-1379. 32779778 10.1002/pon.5434 PMC7323427

[8] Torales J O'Higgins M Castaldelli-Maia JM Ventriglio A. The outbreak of COVID-19 coronavirus and its impact on global mental health. Int J Soc Psychiatr. 2020;66:317-320. 10.1177/0020764020915212 32233719

[9] Seiler A Klaas V Tröster G Fagundes CP. eHealth and mHealth interventions in the treatment of fatigued cancer survivors: a systematic review and meta-analysis. Psychooncology. 2017;26:1239-1253. 28665554 10.1002/pon.4489

[10] Borrelli B Ritterband LM. Special issue on eHealth and mHealth: challenges and future directions for assessment, treatment, and dissemination. Health Psychol. 2015;34S:1205-1208. 26651461 10.1037/hea0000323

[11] Jiang S Liu PL. Digital divide and internet health information seeking among cancer survivors: a trend analysis from 2011 to 2017. Psychooncology. 2020;29:61-67. 31652360 10.1002/pon.5247

[12] Lleras de Frutos M Casellas-Grau A Sumalla EC de Gracia M Borràs JM Ochoa Arnedo C. A systematic and comprehensive review of internet use in cancer patients: psychological factors. Psychooncology. 2020;29:6-16. 31385400 10.1002/pon.5194

[13] Alba-Ruiz R Bermúdez-Tamayo C Pernett JJ Garcia-Gutierrez JF Cózar-Olmo JM Valero-Aguilera B. Adapting the content of cancer web sites to the information needs of patients: reliability and readability. Telemed J E Health. 2013;19:956-966. 24073899 10.1089/tmj.2013.0050 PMC3850662

[14] Matis J Svetlak M Slezackova A Svoboda M Šumec R. Mindfulness-based programs for patients with cancer via eHealth and mobile health: systematic review and synthesis of quantitative research. J Med Internet Res. 2020;22:e20709. 10.2196/20709 PMC7704284 33196452

[15] Nissen ER O'Connor M Kaldo V , et al. Internet-delivered mindfulness-based cognitive therapy for anxiety and depression in cancer survivors: a randomized controlled trial. Psychooncology. 2020;29:68-75. 31600414 10.1002/pon.5237 PMC7004073

[16] Murphy MJ Newby JM Butow P , et al. Randomised controlled trial of internet-delivered cognitive behaviour therapy for clinical depression and/or anxiety in cancer survivors (iCanADAPT Early). Psychooncology. 2020;29:76-85. 31659822 10.1002/pon.5267

[17] Kim SC Shaw BR Shah DV , et al. Interactivity, presence, and targeted patient care: mapping e-Health intervention effects over time for cancer patients with depression. Health Commun. 2019;34:162-171. 29135321 10.1080/10410236.2017.1399504 PMC6158118

[18] van Helmondt SJ van der Lee ML van Woezik RAM Lodder P de Vries J. No effect of CBT-based online self-help training to reduce fear of cancer recurrence: first results of the CAREST multicenter randomized controlled trial. Psychooncology. 2020;29:86-97. 31595627 10.1002/pon.5233

[19] Wagner LI Tooze JA Hall DL , et al. Targeted eHealth intervention to reduce breast cancer survivors' fear of recurrence: results from the FoRtitude randomized trial. J Natl Cancer Inst. 2021;113:1495-1505. 34057469 10.1093/jnci/djab100 PMC8244801

[20] Xu A Wang Y Wu X. Effectiveness of e-health based self-management to improve cancer-related fatigue, self-efficacy and quality of life in cancer patients: Systematic review and meta-analysis. J Adv Nurs. 2019;75:3434-3447. 31566769 10.1111/jan.14197

[21] Faller H Schuler M Richard M Heckl U Weis J Küffner R. Effects of psycho-oncologic interventions on emotional distress and quality of life in adult patients with cancer: systematic review and meta-analysis. J Clin Oncol. 2013;31:782-793. 23319686 10.1200/JCO.2011.40.8922

[22] Jacobsen PB Jim HS. Psychosocial interventions for anxiety and depression in adult cancer patients: achievements and challenges. CA Cancer J Clin. 2008;58:214-230. 18558664 10.3322/CA.2008.0003

[23] Ghanbari E Yektatalab S Mehrabi M. Effects of psychoeducational interventions using mobile apps and mobile-based online group discussions on anxiety and self-esteem in women with breast cancer: randomized controlled trial. JMIR Mhealth Uhealth. 2021;9:e19262. 10.2196/19262 PMC8170553 34003138

[24] González-Fernández S Fernández-Rodríguez C. Acceptance and commitment therapy in cancer: review of applications and findings. Behav Med. 2019;45:255-269. 29558259 10.1080/08964289.2018.1452713

[25] Li H Wong CL Jin X Chen J Chong YY Bai Y. Effects of acceptance and commitment therapy on health-related outcomes for patients with advanced cancer: a systematic review. Int J Nurs Stud. 2021;115:103876. 33517079 10.1016/j.ijnurstu.2021.103876

[26] Li H Wu J Ni Q Zhang J Wang Y He G. Systematic review and meta-analysis of effectiveness of acceptance and commitment therapy in patients with breast cancer. Nurs Res. 2021;70:152-160. 10.1097/NNR.0000000000000499 33492055

[27] Weis J Heckl U. Psychoeducation with cancer patients. Background and scientific evidence. Onkologe. 2021;27:54-62.

[28] Coto-Lesmes R Fernández-Rodríguez C González-Fernández S. Acceptance and commitment therapy in group format for anxiety and depression. a systematic review. J Affect Disord. 2020;263:107-120. 31818766 10.1016/j.jad.2019.11.154

[29] Mathew A Doorenbos AZ Jang MK Hershberger PE. Acceptance and commitment therapy in adult cancer survivors: a systematic review and conceptual model. J Cancer Surviv. 2021;15:427-451. 32949353 10.1007/s11764-020-00938-z PMC10960234

[30] Xiao W Chow KM So WKW Leung DYP Chan CWH . The effectiveness of psychoeducational intervention on managing symptom clusters in patients with cancer: A systematic review of randomized controlled trials. Cancer Nurs. 2016;39:279-291. 26562705 10.1097/NCC.0000000000000313

[31] Corbett TK Groarke A Devane D Carr E Walsh JC McGuire BE. The effectiveness of psychological interventions for fatigue in cancer survivors: systematic review of randomised controlled trials. Syst Rev. 2019;8:324. 31836007 10.1186/s13643-019-1230-2 PMC6911282

[32] Fors EA Bertheussen GF Thune I , et al. Psychosocial interventions as part of breast cancer rehabilitation programs? Results from a systematic review. Psychooncology. 2011;20:909-918. 20821803 10.1002/pon.1844

[33] Stanton AL Ganz PA Kwan L , et al. Outcomes from the moving beyond cancer psychoeducational, randomized, controlled trial with breast cancer patients. J Clin Oncol. 2005;23:6009-6018. 16135469 10.1200/JCO.2005.09.101

[34] Gonzalez M Pascoe MC Yang G , et al. Yoga for depression and anxiety symptoms in people with cancer: a systematic review and meta-analysis. Psychooncology. 2021;30:1196-1208. 33763925 10.1002/pon.5671

[35] Hardoerfer K Jentschke E. Effect of yoga therapy on symptoms of anxiety in cancer patients. Oncol Res Treat. 2018;41:526-532. 30086538 10.1159/000488989

[36] Armer JS Lutgendorf SK. The impact of yoga on fatigue in cancer survivorship: a meta-analysis. JNCI Cancer Spectr. 2020;4:z098. 10.1093/jncics/pkz098 PMC7190209 32368719

[37] Dong B Xie C Jing X Lin L Tian L. Yoga has a solid effect on cancer-related fatigue in patients with breast cancer: a meta-analysis. Breast Cancer Res Treat. 2019;177:5-16. 31127466 10.1007/s10549-019-05278-w

[38] Wayne PM Lee MS Novakowski J , et al. Tai Chi and Qigong for cancer-related symptoms and quality of life: a systematic review and meta-analysis. J Cancer Surviv. 2018;12:256-267. 29222705 10.1007/s11764-017-0665-5 PMC5958892

[39] Zeng Y Xie X Cheng ASK . Qigong or Tai Chi in cancer care: an updated systematic review and meta-analysis. Curr Oncol Rep. 2019;21:48. 30955106 10.1007/s11912-019-0786-2

[40] Zetzl T Renner A Pittig A Jentschke E Roch C van Oorschot B. Yoga effectively reduces fatigue and symptoms of depression in patients with different types of cancer. Support Care Cancer. 2021;29:2973-2982. 33026490 10.1007/s00520-020-05794-2 PMC8062403

[41] Feuerabendt S. Heilkraft Yoga: 100 Übungen für Ihre Gesundheit. Knaur; 2008.

[42] Löwe B Decker O Müller S , et al. Validation and standardization of the Generalized Anxiety Disorder Screener (GAD-7) in the general population. Med Care. 2008;46:266-274. 18388841 10.1097/MLR.0b013e318160d093

[43] Esser P Hartung TJ Friedrich M , et al. The Generalized Anxiety Disorder Screener (GAD-7) and the anxiety module of the Hospital and Depression Scale (HADS-A) as screening tools for generalized anxiety disorder among cancer patients. Psychooncology. 2018;27:1509-1516. 29473255 10.1002/pon.4681

[44] Dankert A Duran G Engst-Hastreiter U , et al. Fear of progression in patients with cancer, diabetes mellitus and chronic arthritis. Rehabilitation. 2003;42:155-163. 12813652 10.1055/s-2003-40094

[45] Herschbach P Berg P Dankert A , et al. Fear of progression in chronic diseases: psychometric properties of the fear of progression questionnaire. J Psychosom Res. 2005;58:505-511. 16125517 10.1016/j.jpsychores.2005.02.007

[46] Mehnert A Herschbach P Berg P Henrich G Koch U. Fear of progression in breast cancer patients–validation of the short form of the fear of progression questionnaire (FoP-Q-SF). Z Psychosom Med Psychother. 2006;52:274-288. 17156600 10.13109/zptm.2006.52.3.274

[47] Weis J Tomaszewski KA Hammerlid E , et al. International psychometric validation of an EORTC quality of life module measuring cancer related fatigue (EORTC QLQ-FA12). J Natl Cancer Inst. 2017;109:djw273. 10.1093/jnci/djw273 28376231

[48] Kroenke K Strine TW Spitzer RL Williams JBW Berry JT Mokdad AH. The PHQ-8 as a measure of current depression in the general population. J Affect Disord. 2009;114:163-173. 18752852 10.1016/j.jad.2008.06.026

[49] Spangenberg L Brähler E Glaesmer H. Identifying depression in the general population - a comparison of PHQ-9, PHQ-8 and PHQ-2. Z Psychosom Med Psychother. 2012;58:3-10. 22427121 10.13109/zptm.2012.58.1.3

[50] Penedo FJ Oswald LB Kronenfeld JP Garcia SF Cella D Yanez B. The increasing value of eHealth in the delivery of patient-centred cancer care. Lancet Oncol. 2020;21:e240-e251. 10.1016/S1470-2045(20)30021-8 PMC7643123 32359500

[51] Tauber NM O'Toole MS Dinkel A , et al. Effect of psychological intervention on fear of cancer recurrence: a systematic review and meta-analysis. J Clin Oncol. 2019;37:2899-2915. 31532725 10.1200/JCO.19.00572 PMC6823887

[52] Christensen H Griffiths KM Farrer L. Adherence in internet interventions for anxiety and depression. J Med Internet Res. 2009;11:e13. 10.2196/jmir.1194 PMC2762797 19403466

[53] Hall DL Luberto CM Philpotts LL Song R Park ER Yeh GY. Mind-body interventions for fear of cancer recurrence: a systematic review and meta-analysis. Psychooncology. 2018;27:2546-2558. 29744965 10.1002/pon.4757 PMC6488231

[54] Taylor S Bellhouse S Allsop M Radford J Yorke J. The role of e-Health in the delivery of care for patients with hematological cancers: a systematic literature review. Telemed J E Health. 2020;26:1093-1105. 32208067 10.1089/tmj.2019.0231

