# Supplementary material for: Effects of a Video Sequence Based Intervention on Anxiety, Fatigue and Depression in Cancer Patients: Results of a Randomized Controlled Trial
Source: Integr Cancer Ther. 2023 Feb 17;22:15347354231153172. doi: 10.1177/15347354231153172 (PMC9940180; doi:10.1177/15347354231153172)
Supplement: sj-docx-1-ict-10.1177_15347354231153172 – Supplemental material for Effects of a Video Sequence Based Intervention on Anxiety, Fatigue and Depression in Cancer Patients: Results of a Randomized Controlled Trial [file sj-docx-1-ict-10.1177_15347354231153172.docx]

**Supplemental Table 1.** Results of the subgroup analysis

|  | **n** | **T1 IG**  **mean (SD)** | **T2 IG**  **mean (SD)** | **Change IG**  **mean (SD)** | **n** | **T1 CG**  **mean (SD)** | **T2 CG**  **mean (SD)** | **Change CG**  **mean (SD)** | **W** | **P-value** |
| --- | --- | --- | --- | --- | --- | --- | --- | --- | --- | --- |
| **GAD7** | 67 | 6.61 (4.58) | 5.72 (4.26) | .90 (3.14) | 68 | 7.00 (5.12) | 6.00 (4.50) | 1.00 (3.71) | 2350 | .751 |
| ***Sex*** |  |  |  |  |  |  |  |  |  |  |
| male | 19 | 4.42 (3.04) | 3.53 (2.50) | .89 (2.21) | 23 | 5.39 (5.11) | 4.78 (3.95) | .61 (3.58) | 243.5 | .526 |
| female | 48 | 7.48 (4.82) | 6.58 (4.51) | .90 (3.47) | 45 | 7.82 (4.98) | 6.62 (4.68) | 1.20 (3.81) | 1070 | .941 |
| ***Therapy goal*** |  |  |  |  |  |  |  |  |  |  |
| curative | 45 | 6.76 (4.99) | 5.80 (4.38) | .96 (3.54) | 48 | 7.42 (5.35) | 6.48 (4.71) | .94 (3.90) | 1070 | .721 |
| palliative | 12 | 7.83 (3.16) | 6.75 (4.49) | 1.08 (2.50) | 12 | 6.00 (4.82) | 5.17 (3.19) | .83 (2.86) | 83 | .54 |
| ***Therapy status*** |  |  |  |  |  |  |  |  |  |  |
| CIT | 39 | 7.21 (4.80) | 6.23 (4.54) | .97 (3.22) | 39 | 5.87 (4.99) | 4.56 (3.69) | 1.31 (3.91) | 753 | .944 |
| CNIT | 27 | 5.78 (4.29) | 4.89 (3.81) | .89 (3.09) | 29 | 8.52 (4.98) | 7.93 (4.83) | .59 (3.45) | 443 | .397 |
| ***Tumor entity*** |  |  |  |  |  |  |  |  |  |  |
| mamma | 22 | 8.54 (4.94) | 6.68 (4.57) | 1.86 (3.93) | 19 | 7.37 (5.74) | 6.47 (4.36) | .89 (4.69) | 262 | .168 |
| hemonc | 18 | 6.00 (5.43) | 4.94 (4.66) | 1.06 (1.89) | 27 | 6.74 (5.23) | 5.67 (5.01) | 1.07 (4.05) | 255.5 | .778 |
| **PA-F-KF** | 56 | 32.43 (9.08) | 31.91 (9.99) | .52 (6.38) | 60 | 33.63 (10.30) | 31.97 (9.68) | 1.67 (5.16) | 1487 | .286 |
| ***Sex*** |  |  |  |  |  |  |  |  |  |  |
| male | 15 | 28.40 (9.01) | 27.40 (10.08) | 1.00 (4.97) | 21 | 31.10 (10.30) | 28.19 (9.06) | 2.90 (6.15) | 133 | .439 |
| female | 41 | 33.90 (8.75) | 33.56 (9.56) | .34 (6.86) | 39 | 35.00 (10.17) | 34.00 (9.49) | 1.00 (4.49) | 724.5 | .472 |
| ***Therapy goal*** |  |  |  |  |  |  |  |  |  |  |
| curative | 40 | 30.98 (8.92) | 30.65 (9.76) | .33 (6.69) | 42 | 34.12 (10.61) | 32.33 (9.53) | 1.79 (4.56) | 710 | .227 |
| palliative | 8 | 40.13 (7.28) | 41.38 (7.31) | -1.25 (4.77) | 9 | 34.11 (11.16) | 34.56 (11.53) | -.44 (6.98) | 37.5 | .923 |
| ***Therapy status*** |  |  |  |  |  |  |  |  |  |  |
| CIT | 31 | 34.68 (9.20) | 33.48 (9.80) | 1.19 (7.19) | 34 | 32.71 (9.63) | 30.82 (9.04) | 1.88 (6.40) | 485.5 | .589 |
| CNIT | 25 | 29.64 (8.27) | 29.96 (10.08) | -.32 (5.21) | 26 | 34.85 (11.20) | 33.46 (10.44) | 1.38 (2.94) | 270.5 | .305 |
| ***Tumor entity*** |  |  |  |  |  |  |  |  |  |  |
| mamma | 17 | 33.47 (7.61) | 32.59 (9.67) | .88 (7.99) | 15 | 35.00 (9.46) | 34.33 (9.08) | .67 (4.84) | 129 | .970 |
| hemonc | 16 | 32.38 (12.41) | 31.56 (11.66) | .81 (7.25) | 27 | 32.85 (10.78) | 30.15 (10.00) | 2.70 (5.67) | 180 | .370 |
| **EORTC QLQ-FA12** | 62 | 36.78 (21.73) | 34.50 (24.65) | 2.28 (19.66) | 70 | 40.60 (23.83) | 37.06 (21.43) | 3.53 (17.70) | 2089.5 | .715 |
| male | 17 | 31.70 (18.58) | 24.84 (23.00) | 6.86 (14.00) | 23 | 38.04 (20.09) | 36.23 (21.34) | 1.81 (17.99) | 237.5 | .254 |
| female | 45 | 38.70 (22.69) | 38.15 (24.51) | .56 (21.30) | 47 | 41.84 (25.57) | 37.47 (21.69) | 4.37 (17.69) | 925 | .301 |
| curative | 41 | 32.52 (21.44) | 31.37 (24.99) | 1.15 (22.39) | 48 | 39.99 (23.44) | 35.53 (21.64) | 4.46 (15.20) | 885.5 | .419 |
| palliative | 11 | 52.53 (19.34) | 46.72 (23.56) | 5.81 (12.45) | 13 | 40.38 (29.61) | 44.87 (21.45) | -4.49 (23.37) | 98 | .13 |
| ***Therapy status*** |  |  |  |  |  |  |  |  |  |  |
| CIT | 36 | 42.44 (20.06) | 38.27 (22.84) | 4.17 (21.94) | 40 | 39.44 (22.14) | 35.56 (20.42) | 3.89 (20.26) | 705.5 | .884 |
| CNIT | 25 | 27.78 (21.47) | 27.44 (25.35) | .33 (16.02) | 30 | 42.13 (26.21) | 39.07 (22.91) | 3.06 (13.89) | 382 | .912 |
| ***Tumor entity*** |  |  |  |  |  |  |  |  |  |  |
| mamma | 20 | 39.03 (19.96) | 31.67 (22.38) | 7.36 (21.43) | 20 | 40.00 (25.27) | 40.14 (23.54) | -.14 (11.63) | 252 | .162 |
| hemonc | 18 | 40.74 (24.40) | 38.58 (29.01) | 2.16 (21.36) | 28 | 40.08 (22.07) | 34.23 (20.80) | 5.85 (17.86) | 223.5 | .527 |
| **PHQ8** | 60 | 6.97 (4.62) | 5.95 (4.85) | 1.02 (3.60) | 65 | 7.69 (5.15) | 6.78 (4.60) | 0.91 (3.56) | 1938 | .954 |
| male | 18 | 5.28 (2.95) | 3.72 (3.01) | 1.56 (3.40) | 23 | 7.13 (5.34) | 6.30 (5.15) | .83 (3.60) | 234.5 | .475 |
| female | 42 | 7.69 (5.03) | 6.90 (5.20) | .79 (3.69) | 42 | 8.00 (5.09) | 7.05 (4.31) | .95 (3.58) | 810 | .519 |
| ***Therapy goal*** |  |  |  |  |  |  |  |  |  |  |
| curative | 39 | 6.49 (4.97) | 5.82 (4.86) | .67 (3.33) | 46 | 8.22 (5.40) | 6.89 (4.88) | 1.33 (3.83) | 751.1 | .197 |
| palliative | 11 | 8.45 (4.46) | 7.73 (5.69) | .73 (4.05) | 11 | 7.00 (4.63) | 7.55 (3.50) | -.55 (2.66) | 74.5 | .372 |
| ***Therapy status*** |  |  |  |  |  |  |  |  |  |  |
| CIT | 36 | 7.89 (4.13) | 6.56 (4.82) | 1.33 (4.01) | 36 | 7.17 (4.85) | 6.17 (4.88) | 1.00 (3.62) | 665 | .852 |
| CNIT | 23 | 5.30 (4.98) | 4.57 (4.36) | .74 (2.77) | 29 | 8.34 (5.52) | 7.55 (4.69) | .79 (3.55) | 323 | .851 |
| ***Tumor entity*** |  |  |  |  |  |  |  |  |  |  |
| BC | 19 | 7.63 (4.46) | 6.16 (5.12) | 1.47 (4.01) | 17 | 7.71 (4.73) | 7.35 (4.50) | .35 (3.35) | 198 | .246 |
| hemonc | 18 | 7.39 (4.84) | 5.72 (4.79) | 1.67 (3.20) | 27 | 6.70 (4.89) | 6.22 (4.79) | .48 (3.20) | 286 | .321 |

Abbreviations: n, number of patients; T1, baseline; T2, after the intervention or waiting period; IG, intervention group, CG, control group; SD, standard deviation; CIT, currently in therapy; CNIT, currently not in therapy; BC, breast cancer; hemonc, hemato-oncological tumors
